# Supplementary material for: Agroforestry adoption and its influence on soil quality under smallholder maize production systems in western Kenya
Source: PLoS One. 2025 Apr 8;20(4):e0313385. doi: 10.1371/journal.pone.0313385 (PMC11991676; doi:10.1371/journal.pone.0313385)
Supplement: Attachment — Submitted filename: Response_to_Reviewers_auresp_2.docx [file pone.0313385.s007.docx]

**School of Agriculture and Biotechnology	February 5^th^, 2025**

University of Eldoret

P.O.BOX 1125-30100

Eldoret, Kenya

<http://www.uoeld.ac.ke>

Tel:+254(0) 774 249 552

Dear Editors

We thank the editorial team for effectively taking this manuscript through the various stages of review aimed at improving the overall quality of the manuscript. Herewith attached is a response to the editor’s comments and a revised version of the manuscript addressing key concerns.

We are confident that the revised version of the manuscript is greatly improved, and we hope that it can be considered for publication in PLOS ONE Journal.

Henry Tamba Nyuma (PhD Candidate)

Researcher

Department of Soil Science

School of Agriculture and Biotechnology

University of Eldoret

On behalf of all authors.

**Editor’s comments**

**Comment 1:** Include full names of all authors.

**Response:** The full names of the authors and co-authors are included in the list of authors as requested.

**Comment 2:** Check if the statement is correct on Line 59

**Response:** The statement has been checked as per the cited reference, that soil moisture interacts with hydrated cations on the exchange sites, hence affecting moisture retention

**Comment 3:** insert citations in statements on lines 170-179.

**Response:** Citations inserted on lines 170-179 as required

**Comment 4:** Please use (a), (b) and (c) for the individual figures

**Response:** Letters inserted for individual figures throughout the manuscript as required.

**Comment 5:** Remove these charges from the cations throughout the manuscript

**Response:** Charges were removed from all cations throughout the manuscript as requested

**Comment 6:** This statement is on line 447

**Response:** The statement has been completed and adjusted

**Comment 7:** check and revise the list of references

**Response:** The list of references was re-checked and revised according to the requirements of the PLOS One Journal.
