# Supplementary material for: Agroforestry adoption and its influence on soil quality under smallholder maize production systems in western Kenya
Source: PLoS One. 2025 Apr 8;20(4):e0313385. doi: 10.1371/journal.pone.0313385 (PMC11991676; doi:10.1371/journal.pone.0313385)
Supplement: Attachment — Submitted filename: agroforestry and soil quality.docx [file pone.0313385.s002.docx]

**Agroforestry adoption and its influence on soil quality under smallholder maize**

**production systems in western Kenya**

Henry Tamba Nyuma

Comment: Include Ruth Njoroge and Abigael Otinga in the author list

**ABSTRACT**

Comment: Not complete. You stopped at results. No discussion, conclusion and recommendation. Please populate this abstract

**INTRODUCTION**

Expand your introduction by discussing lessons drawn globally, nationally then lastly in the study area before opening up to what you are investigating

**METHODOLOGY**

- Line 94: explain unique environmental characteristics that qualify the choice of the study area
- State your how you selected your ‘n’ and why the approach you chose is superior

**RESULTS and DISCUSSION**

Results well presented for analyzed nutrients

Interpretation well done under discussion

Comparison of results with previous studies well done

**CONCLUSION**

Well done
