# Supplementary material for: Agroforestry adoption and its influence on soil quality under smallholder maize production systems in western Kenya
Source: PLoS One. 2025 Apr 8;20(4):e0313385. doi: 10.1371/journal.pone.0313385 (PMC11991676; doi:10.1371/journal.pone.0313385)
Supplement: Attachment — Submitted filename: Response to Reviewers.docx [file pone.0313385.s006.docx]

**School of Agriculture and Biotechnology	December 17^th^,2024**

University of Eldoret

P.O.BOX 1125-30100

Eldoret, Kenya

<http://www.uoeld.ac.ke>

Tel:+254(0) 774 249 552

Dear Editors

We write to express thanks to the reviewers for their generous comments on the manuscript and herewith attached is a response to the reviewers’ comments, and an edited version of the manuscript addressing their concerns. In particular, the abstract, method of sample selection, method of data analysis, citations, and grammatical errors have been revised or addressed as recommended by the reviewers.

We are confident that the revised version of the manuscript is now suitable for publication in PLOS ONE journals.

Henry Tamba Nyuma (PhD Candidate)

Researcher

Department of Soil Science

School of Agriculture and Biotechnology

University of Eldoret

On behalf of all authors.

**Response to Reviewers’ comments**

Reviewer #1

Comment: Include Ruth Njoroge and Abigael Otinga in the author list. As project supervisors, they have contributed immensely to the manuscript.

The co-authors mentioned above have already been included in the author list.


ABSTRACT

Comment: This is Not complete. You stopped at results. No discussion, conclusion and recommendation is summarized within the abstract. Please populate this abstract to make it informative.

The Abstract has been fully revised to include a discussion, conclusion, and recommendations as requested by the reviewers.

INTRODUCTION
Expand your introduction by discussing lessons drawn globally, nationally then lastly in the study area before opening up to what you are investigating. It’s important to open up the introduction to broaden your scope for investigation.

A global, regional, and national perspective of agroforestry has been added in the introduction section.
METHODOLOGY

λLine 94: explain unique environmental characteristics that qualify the choice of the study area

In line 94, the choice of the study area was based on the earlier introduction of agroforestry, declining soil fertility and similarly in land use practices, and agroecology has been included in the revised version.
λState how you selected your ‘n’ and why the approach you chose is superior to other statistical approaches on population selection and choice of n

The sample size was determined as a result of the adoption of agroforestry practices in the study area, supported by two reconnaissance visits in close consultation with county agricultural officers.

Reviewer #2: Abstract:
Line 10: was-introduced? Why a hyphen?

The hyphen has been removed and the sentences have been rectified.
Line 24-25: Can Sesbania and Leucaena influence Clay? How?

The intent was to highlight the effects of agroforestry tree species on the physical characteristics of soils in the study, with clay content and bulk density (BD)being key indicators of soil physical fertility. However, given that the study did not include soil moisture retention, which is mainly influenced by particle size, the concerns by Reviewer #2 were considered and addressed in regard to the influence of agroforestry adoption on soil clay content.
Introduction:
Line 38: 36–75 billion hectares? please check the value.

Yeah, this might have been an overestimation of the extent of soil degradation through erosion, but the appropriate figures and citations have been rectified.
Line 49-50: showed a 30-60% decline in the yields of maize ? English may be improved here

Grammar has been improved in the corrected version of the manuscript.

Overall, the introduction section is written meticulously

Methods:

Line 87-88: Figure 1 should be put in bracket? Please see journal style and apply throughout the manuscript.

Journal style regarding the citation of figures has been consulted, and all citations of “Figures” have been changed to “Fig” as recommended.
Line 106-118: Please mention information on soil sampling depth in this section

Soil sampling depth ( 0-30 cm ) within 1 meter radius of agroforestry trees is included in the methodology section.
Line 124: correct English
Grammar was checked as recommended.
Section 2.4 Data Analysis: Please explain how one-way ANOVA was conducted with Paired observations? Or it was done between agroforestry tree species. Please mention clearly.

The use of one-way ANOVA was for the effects of agroforestry tree species on soil characteristics, while pair test comparisons were done for adoption practices and study locations are mentioned in the data analysis section.

Results:
Line 180: should one put clay content as a variable to be affected by agroforestry practices? Also in Table 2? Please check throughout the manuscript and modify if required

This has been addressed throughoutut the manuscript as recommended by the reviewer.
Discussion:
Line 280: also water erosion?

This has been addressed.
Line 280-281: agree with [45] who reported….please check journal format..and change throughout the manuscript accordingly.
Line 302: can be is increased? Correct the same.

Addressed as recommended
Line 309: Suleiman [60]? Refer to comment line 280-281 above.
Line 327: Riyadh et al. ([68]?

Citation style guidelines have been followed and references are appropriately cited in line with PLOS One citation style.
The discussion section is fairly well written and can be improved with improvement in English.

Conclusion: Some important results may be highlighted in this section, otherwise well written.

Additional information has been added to the conclusion section as well as recommended.
